# Dexamethasone induces p21^cip1/waf1^ expression via FoxO3a independently of the Lamin A/C‐HDAC2 interaction in Ataxia Telangiectasia

**DOI:** 10.1002/2211-5463.13663

**Published:** 2023-07-03

**Authors:** Anastasia Ricci, Federica Biancucci, Gianluca Morganti, Mauro Magnani, Michele Menotta

**Affiliations:** ^1^ Department of Biomolecular Sciences University of Urbino ‘Carlo Bo’ Italy

**Keywords:** Ataxia‐Telangiectasia, ATM, CDKN1A, dexamethasone, Lamin A/C, p21

## Abstract

Ataxia‐Telangiectasia (A‐T) is a very rare autosomal recessive multisystemic disorder which to date is still uncurable. The use of glucocorticoid analogs, such as dexamethasone (dex), can improve neurological symptoms in patients, but the molecular mechanism of action of these analogs remains unclear. Here, we report the effects of dex in regulating the interaction between Lamin A/C and HDAC2 in WT and A‐T cells. Upon administration of dex to A‐T cells, we first observed that the accumulation of HDAC2 on the *CDKN1A* promoter did not exert a repressive role on p21^cip1/waf1^ expression, and second, we established that HDAC2 accumulation was not dependent on Lamin A/C. Both of these results are contrary to previous reported outcomes in other cellular models. Furthermore, large amounts of LAP2α and FoxO3a were found to occupy the *CDKN1A* promoter with matched p21^cip1/waf1^ overexpression. Hence, in A‐T cells p21 could be activated as a result of a dex‐induced rearrangement of a multicomponent complex, composed of Lamin A/C, HDAC2, LAP2α, pRb, E2F1, and FoxO3a, at the *CDKN1A* gene promoter.

## Graphical abstract

Ataxia Telangiectasia (A‐T) is a rare genetic disease caused by ATM mutations. In A‐T cells treated with dexamethasone, a drug employed in the clinic to alleviate symptoms, the expression of p21^cip1/waf1^ is independent of HDAC2 accumulation on the *CDKN1A* promoter, which is discordant with outcomes previously reported in other cellular models.

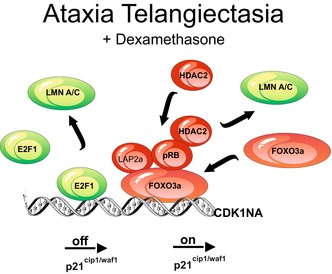

**Abbreviations**
A‐T: Ataxia Telangiectasia
ATM: Ataxia Telangiectasia mutated
*CDKN1A*
: Cyclin‐dependent kinase inhibitor 1A
Co‐IP: Co‐immunoprecipitation
Dex: Dexamethasone
E2F1: E2F transcription factor 1
FoxO3a: Forkhead box O3
HDAC2: Histone deacetylase 2
Lap2α: Lamin‐ associated polypeptide 2 alpha
LMN A/C: Lamin A/C
PLA: Proximity ligation assay
pRb: Retinoblastoma
THBS1: Thrombospondin 1
WLN: Whole lane normalization
WT: Wild‐type

Ataxia Telangiectasia (A‐T) is a rare genetic syndrome caused by biallelic mutations in the Ataxia Telangiectasia Mutated (ATM) gene [1] that codes for a protein kinase of the same name, belonging to the PI3 kinase‐like kinase [2]. The loss of the ATM protein leads to pleiotropic clinical phenotypes [3] as it is involved in many different molecular mechanisms [4]. The most important and best‐understood roles of ATM are nuclear functions such as DNA repair and cell cycle control. Consequently, the loss of ATM function is responsible for the aberrant proliferation of cells due to the unrepaired double‐strand DNA breaks, increasing the risk of cancer and radiosensitivity. Evidence is now emerging that ATM also plays a role in oxidative stress, mitochondrial dysfunction [5, 6, 7], vesicles dynamics, autophagy, mitophagy, and peroxyphagy [8, 9, 10]. Unfortunately, there is no cure currently available for A‐T patients, but only supportive therapies which aim to slow neurodegeneration, counteract immunodeficiency, and prevent the onset of lymphoid tumors. The administration of glucocorticoids has been proven to improve neurological symptoms and the overall quality of life of A‐T patients, in both observational studies and clinical trials [11, 12, 13]. Therefore, investigations have been carried out to elucidate the mechanism of action of glucocorticoids in A‐T cellular models, or where possible, in biological samples from patients, revealing that they can specifically modulate several cellular functions, namely splicing, gene and protein expression, metabolism, red‐ox homeostasis, and autophagy [10, 14, 15, 16, 17, 18, 19, 20]. Furthermore, we recently described the role of dexamethasone in Lamin A/C homeostasis, proving that A type Lamins might be involved in the Ataxia Telangiectasia pathology [19]. Leading on from this work, preliminary Lamin A interactomes were carried out using co‐immunoprecipitation (Co‐IP) experiments. The results of these interactomes revealed that the well‐known Lamin A/C‐HDAC2 interaction is modulated by dex. This interaction is critical as it leads to the recruitment of HDAC2 on the CDKN1A promoter, causing histone deacetylation and p21^cip1/waf1^ (p21) downregulation [21]. Furthermore, it was found that Lamin A/C‐HDAC2 interaction is decreased in Hutchinson–Gilford Progeria syndrome, leading to p21 upregulation [21]. This can contribute to impaired cell cycle regulation and accelerated senescence, all features which are also present in A‐T. Hence, we performed a parallel HDAC2‐Lamin A/C characterization in an A‐T cellular model, expanding the investigations also to other Lamin A/C partners, LAP2α, pRb, and E2F1 previously identified as dex modulated [19], which could contribute to the regulation of p21 expression.

In the present study, we report the response of A‐T cells compared with wild‐type (WT) cells after dex treatment in regulating p21 expression by the rearrangement of Lamin A/C‐HDAC2 in a multicomponent complex.

## Methods

### Cell lines, culture conditions, and drug administration

The hTERT immortalized fibroblasts WT AG09429 hTERT (WT hT) and AT GM00648 hTERT (AT 648 hT) were obtained as described previously [18], and grown in MEM (Eagle formulation). The medium was supplemented with 2 mmol·L^−1^ l‐glutamine, 100 U·mL^−1^ penicillin, and 0.1 mg·mL^−1^ streptomycin (Sigma‐Aldrich, St. Louis, MO, USA), with the addition of 10% fetal bovine serum (Thermo Fisher Scientific, Waltham, MA, USA) and 10 mm glucose. All cells were maintained at 37 °C with 5% CO_2_ and treated with 100 nm dex for 72 h prior to each analysis. Dimethylsulfoxide was used as the drug vehicle and thus was administered in untreated cells as the control.

### Antibodies

The following list of antibodies were used in the current study: anti‐Lamin A/C (Cell Signaling Technology CST #2032, Danvers, MA, USA and Diatheva Srl #ANT0072, Cartoceto, PU, Italy), FITC‐conjugated anti‐Lamin A/C (CST #8617), anti‐LAP2α (BETHYL, Laboratories, Montgomery, TX, USA, A304‐839A‐M) anti‐pRB (CST #9309, BETHYL #A302‐433A‐T), anti‐E2F1 (Santa Cruz Biotechnology Inc., Santa Cruz, CA, USA, #sc‐251, BETHYL #A300‐766A‐M), anti‐HDAC2 (CST #5113), and anti‐FoxO3a (CST #2497). HRP‐conjugated secondary antibodies used for western blotting were purchased from Bio‐Rad (Hercules, CA, USA), while fluorescence‐conjugated secondary antibodies were purchased from Merck–Millipore (Burlington, MA, USA).

### Western blotting

Total proteins were extracted using the Protein Extraction Reagent Type 4 (P4, Sigma‐Aldrich). Cells were sonicated with 10 pulses of 15 s at 45 W Labsonic 1510 Sonicator (Braun, Melsungen, Germany) and clarified by centrifugation for 10 min at 10 000 *g*. Protein concentration was determined by the Bio‐Rad Protein Assay, based on Bradford's method. Twenty micrograms of proteins were separated by SDS/PAGE (Novex Tris‐Glycine gels) according to the Laemmli protocol [22] and then transferred to nitrocellulose (0.22 μm; Bio‐Rad) or LF PVDF (0.45 μm; Bio‐Rad) by wet transfer and Towbin blotting buffer (50 mm Tris, 150 mm NaCl, 20% v/v methanol). Membranes were probed with the primary antibodies diluted in 5% w/v nonfat dry milk or 5% BSA in TBS‐T. The primary antibodies used in this study were anti‐HDAC2 (CST) and anti‐Lamin A/C (CST). The utilized secondary antibodies were Alexa Fluor 790 (Thermo Fisher Scientific). Immunoreactive bands were recorded using the enhanced chemiluminescence (Advansta, Menlo Park, CA, USA) or fluorescence acquisition by ChemiDoc Touch Imaging System (Bio‐Rad). The whole lane normalization (WLN) strategy was adopted in all western blot analyses using a trihalo compound for protein visualization [23, 24, 25]. Acquired images were analyzed by image lab software 5.2.1 (Bio‐Rad) [26].

### Lamin A/C co‐immunoprecipitation

Co‐immunoprecipitation of nuclear protein fractions was performed using standard methods. Briefly, cells were lysed in cytosolic lysis buffer (10 mm HEPES, pH 7.5, 1.5 mm MgCl_2_, 10 mm KCl, 10% glycerol, 0.2% NP‐40, 1 mm DDT, and protease inhibitors) in ice for 10 min. After centrifugation, the nuclei pellet was lysed in nuclear lysis buffer (10 mm HEPES, pH 7.5, 1.5 mm MgCl_2_, 300 mm KCl, 10% glycerol, 0.2% NP‐40, 1 mm DTT protease inhibitors), and sonicated with three pulses of 5 s at 50 Watts Labsonic 1510 Sonicator (Braun, Melsungen, Germany) and clarified by centrifugation for 10 min at 10 000 RCF. One hundred micrograms of nuclear protein were immunoprecipitated with anti‐LMN A/C antibody (1 : 100 CST or Diatheva) in nuclear lysis buffer at a final concentration of 150 mm KCl. MOCK sample was prepared as control. Immuno‐precipitates were incubated with Protein A/G agarose beads, at 4 °C for 4 h. Agarose beads were copiously washed in wash buffer (10 mm HEPES, pH 7.5, 1.5 mm MgCl_2_, 150 mm, KCl, 0.25% NP‐40, and 10% glycerol). Immunoprecipitated protein complexes were directly boiled in Laemmli's buffer and subjected to western blot analysis as described previously using anti‐HDAC2 (CST).

### Proximity ligation assay

Protein interaction detection was performed using the Duolink system (Sigma‐Aldrich) according to the manufacturer's instructions. Cells were seeded in Lab‐Tek II chamber slides (NUNC). After fixing for 10 min with 4% formaldehyde and then with 100% cold methanol, they were permeabilized by 0.5% NP‐40 in PBS for 10 min. After performing the blocking procedure for 1.5 h at room temperature, primary antibodies were applied. The antibody specificity setup was determined by examining the experimental control outputs at different antibody dilutions. Once each optimum condition was found, the proximity ligation assay (PLA) experiments were carried out. Nuclei were stained with 4′,6‐diamidino‐2‐phenylindole at a final concentration of 0.2 μg·mL^−1^ or were highlighted by FITC anti‐Lamin A/C adopting the approach indicated by the manufacturer Duolink. Signals were analyzed by imagej (NIH, Bethesda, MD, USA) using nuclear ROI and subtracting the average background.

### p21 gene expression

Total RNA was extracted from WT hT and AT 648 hT fibroblast cell lines treated with dex or not treated using the RNeasy mini kit (QIAGEN, 74104 Valencia, CA, USA). Five hundred nanograms of RNA were employed in each experiment to obtain cDNA PrimeScript™ RT Master Mix (Takara, Bio, Shiga, Japan). One nanogram of cDNA was used in each PCR reaction for TaqMan Gene Expression Assays (Thermo Fisher Scientific) of PPIC and PPIA as housekeeping genes. The target gene p21 expresion was evaluated by using the SYBR Green Premix Ex Taq Tli RNase H Plus (Takara) in combination with the primers 5′‐TGGAGACTCTCAGGGTCGAAAA‐3′ and 5′‐TTCCTGTGGGCGGATTAGG‐3′. The efficiency of the reaction was determined by standard curves (on average 97% efficiency), and the final standard dilution was sample CT inclusive. Amplification plots were analyzed using the ABI PRISM 7500 sequence detection system (Applied Biosystems, Waltham, MA, USA), and the relative DNA amounts were calculated by the 1/2^Δ^ ^Ct^ method.

### ChIP followed by qPCR

ChIP was performed for each culture condition. Briefly, cells were crosslinked for 10 min with 1% formaldehyde and the nuclei were prepared by cell lysis buffer (5 mm HEPES‐KOH pH 7.5, 85 mm KCl, 0.5% NP‐40 1× complete protease inhibitor, 10 min in ice). Nuclei‐containing pellets were resuspended in lysis buffer (50 mm Tris–HCl pH8, 10 mm EDTA, 1% SDS, 1× complete protease inhibitor) and subsequently sonicated by Bioruptor Plus for 12–16 cycles in order to obtain a comparable fragment size range among the samples, between 100 and 600 bp. Fifty micrograms of input chromatin was diluted in binding buffer (Final: 0.2% SDS, 1% Triton X100, 150 mm NaCl, 2 mm EDTA, 0.5 mm EGTA, 10 mm Tris pH 8.5 1× complete protease inhibitor) and incubated with the antibodies: HDAC2, Lamin A/C, E2F1, Lap2α, pRB, and FoxO3a. MOCK samples were prepared as controls. Complexes were purified with A/G beads, and after washing, chromatin was de‐crosslinked, RNAase A and proteinase K were added, and DNA was purified. The ChIP was repeated in triplicate. The obtained purified DNAs were amplified by qPCR using the SYBR Green Premix Ex Taq Tli RNase H Plus (Takara). The employed primers, surrounding both the FoxO3a and E2F1 binding site in the *CDKN1A* promoter, were F1 5′‐ATGCTAGGAACATGAGCAAACTG‐3′ and R1 5′‐GCCAGAAAGCCAATCAGAGC‐3′. The qPCRs efficiency was established by standard curves (on average 95% efficiency), ensuring that the last standard dilution was sample CT inclusive and the relative DNA amounts were calculated by the 1/2^Δ^ ^Ct^ method.

### Statistical analysis

graphpad prism (San Diego, CA, USA) was used for statistical analyses and graph generation. Statistical tests were chosen according to sample size and variance homogeneity. The statistical tests used were the Friedman test followed by Dunn's test and Kruskal–Wallis test followed by Dunn's test. Medians were considered statistically different with *P* ≤ 0.05.

## Results

**Fig. 1:**
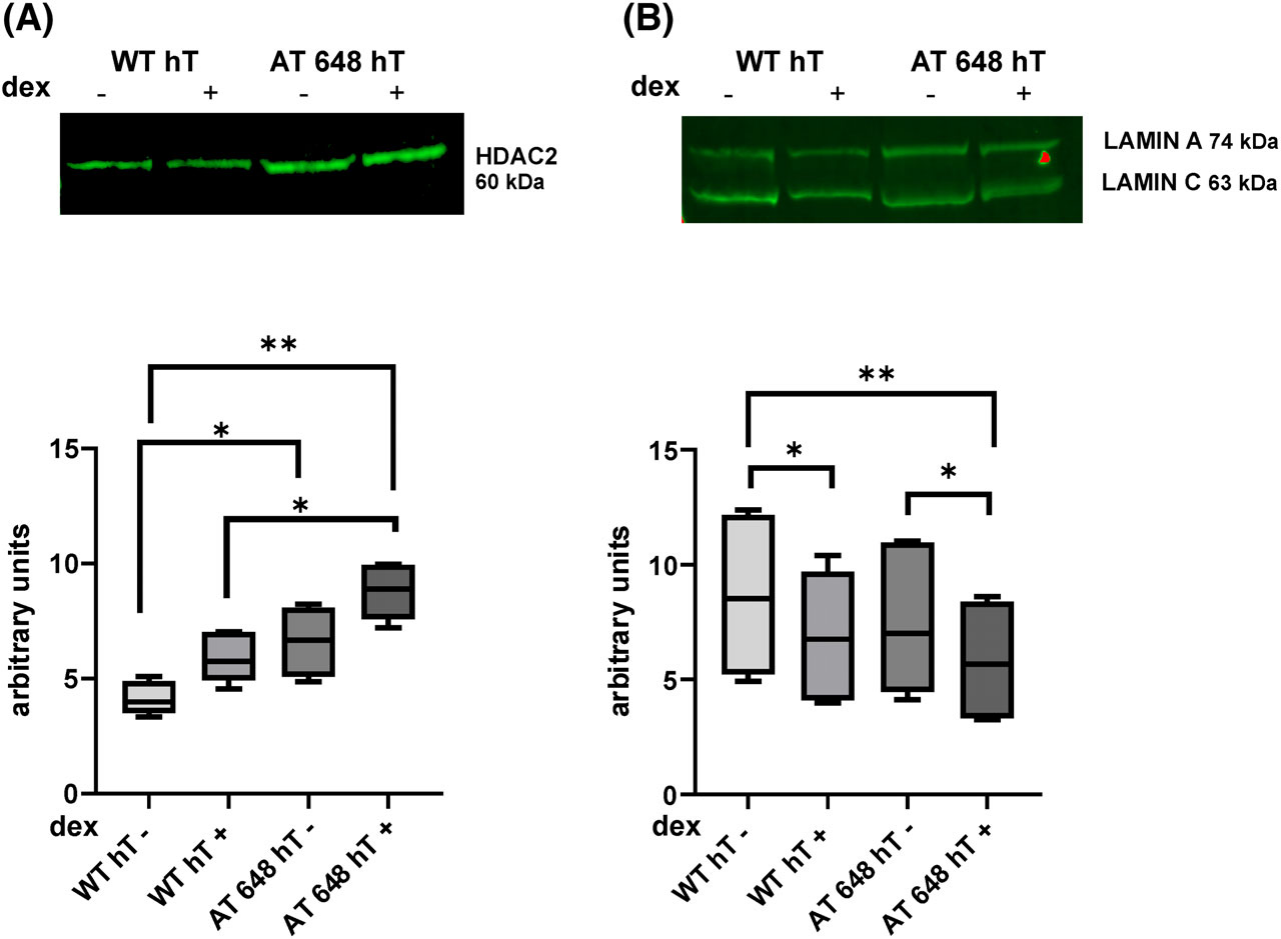
Western blot representation of total (A) HDAC2 and (B) Lamin A/C in WT hT and AT 648 hT cells. AT 648 hT cells showed a greater amount of total HDAC2 than WT hT, while dex did not change HDAC2 total content in both samples. Regarding Lamin A/C content, WT hT and AT 648 hT cells presented the same amount, and a reduction in the protein after dex treatment was observable in both cell lines (Friedman test followed by Dunn's test, *n* = 7, *P < 0.05, **P < 0.01). The WLN strategy was adopted using the stain‐free method for protein visualization and normalization.

The previous preliminary data from a proteomic study of the dex‐modulated interactome of Lamin A/C in A‐T cells (data not shown) suggested HDAC2 as a potential partner. Due to HDAC2's potential role in regulating p21 expression by Lamin A/C [21], we decided to investigate further. Firstly, we evaluated the total amount of Lamin A/C and HDAC2 in WT hT and AT 648 hT cells, and their possible modulation after dex treatment (Fig. 1). Without dex treatment, a greater amount of HDAC2 was found in AT 648 hT cells compared with levels found in WT cells. After stimulation with dex, the total content of HDAC2 remained unchanged in both samples. Conversely, before administration of dex, AT 648 hT cells presented the same protein level of Lamin A/C as WT cells but after treatment with dex the levels were reduced in both cell lines.

**Fig. 2:**
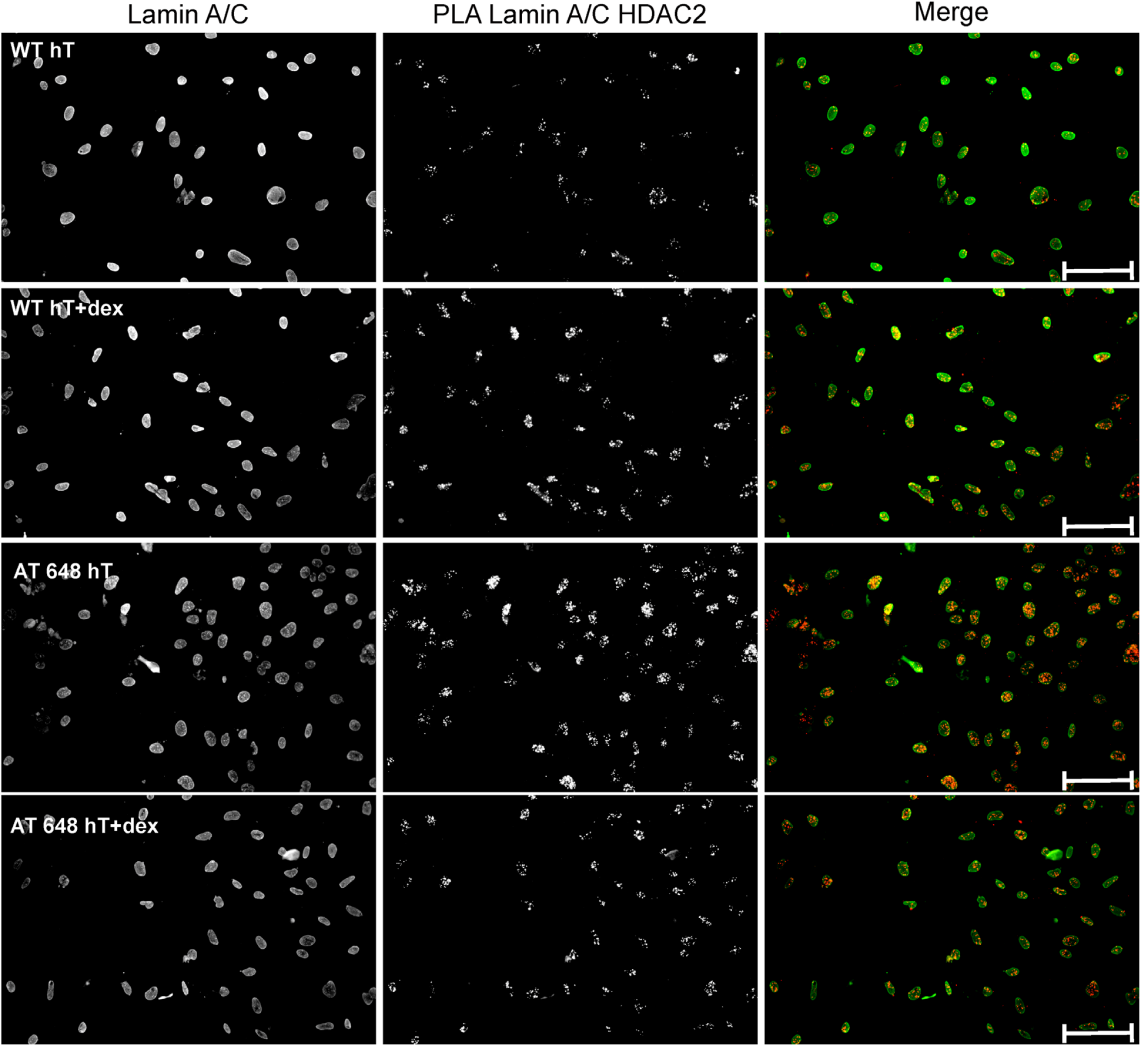
Proximity ligation assay to test Lamin A/C‐HDAC2 interaction modulation by dex. WT hT and AT 648 hT cells were treated or not with dex for 72 h prior to performing the PLA procedure. A strong interaction signal was highlighted especially in AT 648 hT cells, and dex could modulate it as reported in Fig. 3 (scale bar 10 μm).

**Fig. 3:**
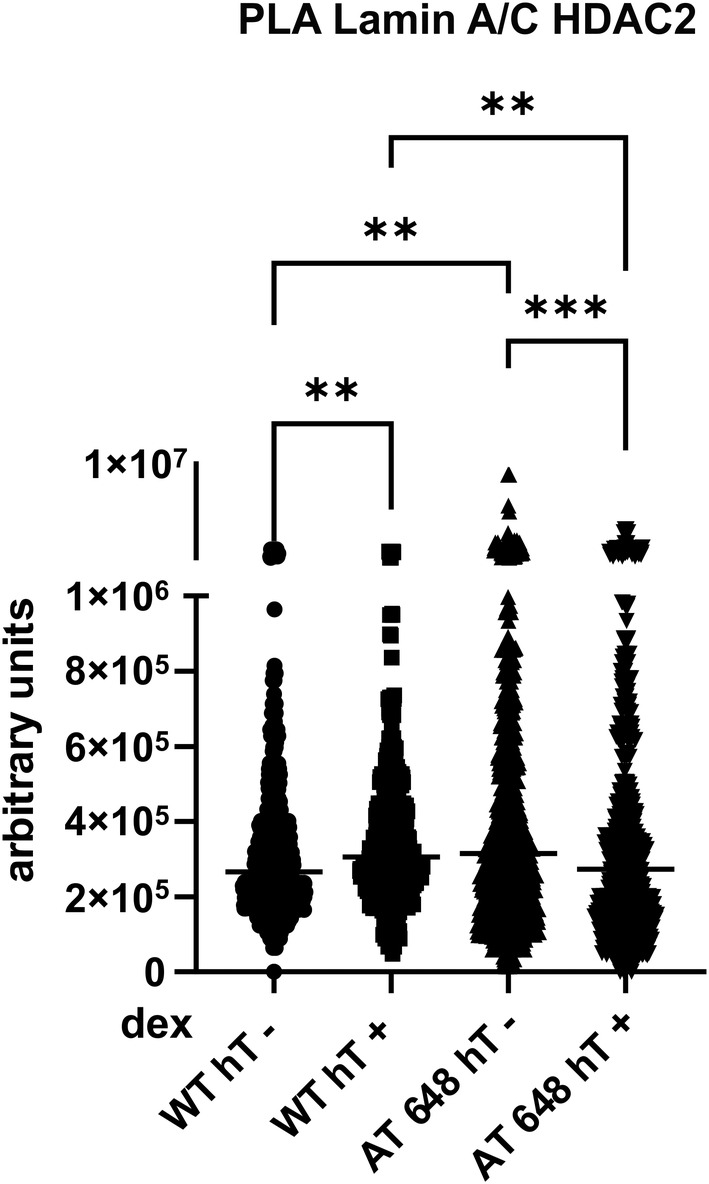
Quantitation of PLA signals. Signals from nuclear ROI were processed and plotted as illustrated. The Lamin A/C‐HDAC2 interaction was statistically enhanced by dex in WT hT cells, while it was decreased by the treatment in AT 648 hT cells, diminishing it to the level found in WT hT cells at basal condition (Kruskal–Wallis test followed by Dunn's multiple comparisons test *n* = 5, **P < 0.01, ***P < 0.001).

**Fig. 4:**
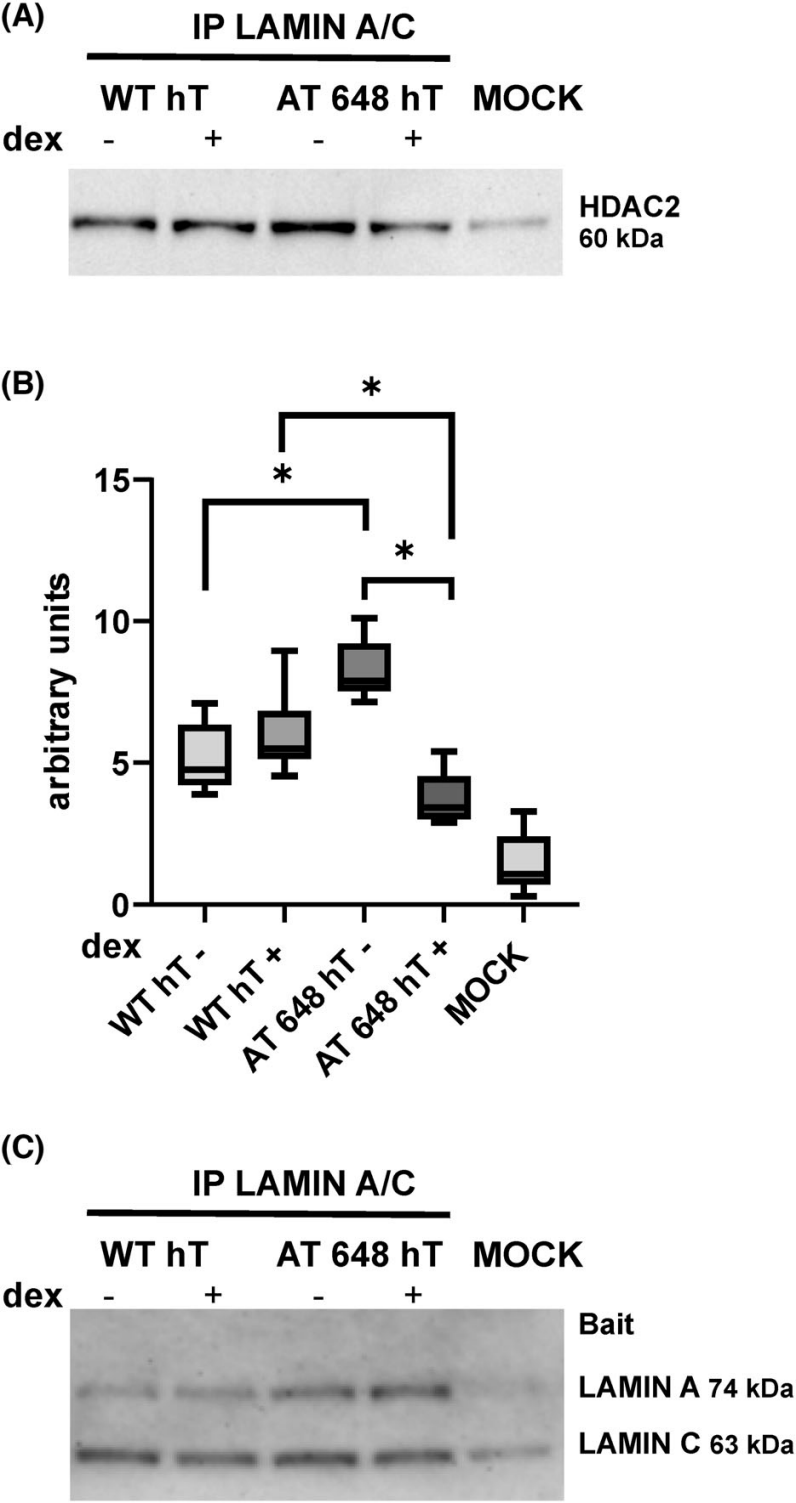
(A) Representative image and (B) quantification of western blot of HDAC2 probed membranes containing Lamin A/C co‐immunoprecipitated proteins. Dex decreased the interaction between Lamin A/C and HDAC2 in AT 648 hT cells, bringing it to the level found in untreated WT hT cells and confirming PLA assay results (Friedman test followed by Dunn's test, *n* = 5, *P < 0.05). (C) The amount of bait Lamin A/C discovered in Co‐IP.

The modulation of Lamin A/C‐HDAC2 interaction by dex was then assayed and confirmed by PLA and by the Co‐IP technique. In Fig. 2, a representative image of the PLA output of WT hT and AT 648 hT cells is shown with and without dex treatment. The differences between the two samples are quantified and reported in Fig. 3. The first emerging evidence is the greater amounts of recorded interactions in AT 648 hT cells compared with WT ones at basal condition. Dex administration in A‐T could decrease the level of interaction to that found in WT (no statistical difference between WT hT and dex‐treated AT 648 hT cells was observed). This behavior was also confirmed by Co‐IP assay, as reported in Fig. 4: AT 648 hT had more Lamin A/C and HDAC2 interactions in comparison with WT hT cells, and the treatment with dex reduced them to untreated WT levels. Unlike PLA assay, no modulation was statistically recorded in WT hT cells after dex stimulation.

**Fig. 5:**
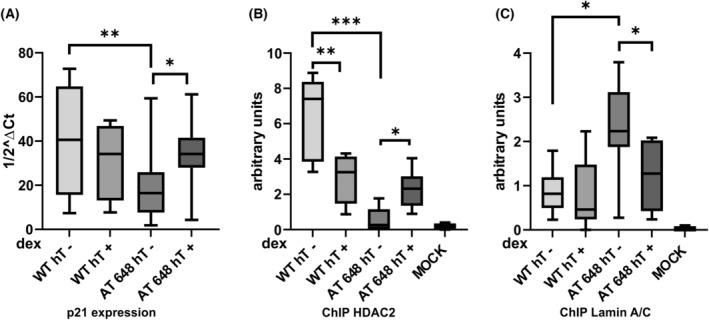
(A) Expression level of p21, ChIP of (B) HDAC2 on the *CDKN1A* promoter, and of (C) Lamin A/C. p21 expression was lower in AT 648 hT than WT hT cells and dex could increase it only in AT 648 hT cells. A low amount of HDAC2 on the *CDKN1A* promoter was noticed in AT 648 hT cells, and dex increased its quantity. A larger quantity of Lamin A/C cells is localized on *CDKN1A* promoter in AT 648 hT cells, while dex diminished its amount. In WT hT, the amount of Lamin A/C was unaffected by dex (Friedman test followed by Dunn's test, *n* = 5, *P < 0.05, **P < 0.01, ***P < 0.001).

Since HDAC2‐Lamin A/C interaction is involved in p21 expression [21], the quantitative amounts of p21 messengers were assayed and ChIP of both proteins on the *CDKN1A* promoter was performed, as illustrated in Fig. 5. p21 expression was lower in AT 648 hT cells than in WT hT cells at basal conditions, while dex restored its quantity at comparable level of WT cells (Fig. 5A). This is consistent with the lack of ATM‐p53‐p21 axis in AT 648 hT cells, but the analysis of *CDKN1A* promoter occupancy by Lamin A/C and HDAC2 was conflicting with the previous results reported in other cell lines [21]. In fact, at basal conditions, the amount of HDAC2 in the FoxO3a binding site of p21 promoter was lower in AT 648 hT cells, which also presented a lower p21 expression, compared with WT hT cells, while a larger amount of Lamin A/C was observed in the same cells compared with WT ones (Fig. 5B,C). Dex decreased HDAC2 presence in WT hT and increased it in AT 648 hT cells and strongly affected Lamin amounts, decreasing it, only in AT 648 hT cells.

**Fig. 6:**
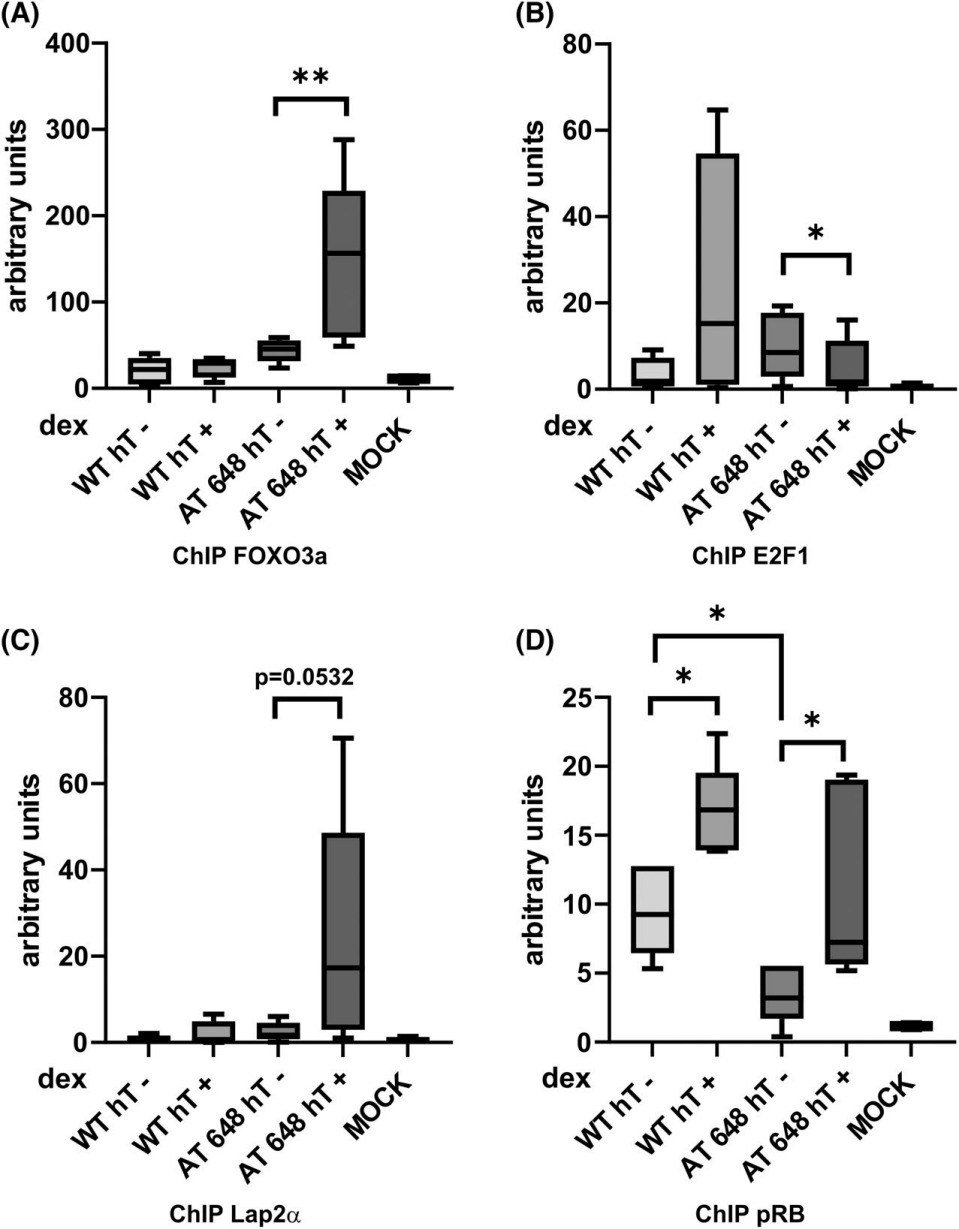
ChIP of (A) FoxO3a, (B) E2F1, (C) Lap2a and (D) pRB. Dex was capable of specifically modulating the amounts of FoxO3a, E2F1, and Lap2α on the *CDKN1A* promoter only in AT 648 hT cells, while pRB was dex affected also in WT hT samples on the *CDKN1A* promoter. Additionally, pRB is expressed at a very low level in untreated AT 648 hT cells (Friedman test followed by Dunn's test, *n* = 5, *P < 0.05, **P < 0.01).

We have therefore highlighted a discrepancy in the roles of HDAC2 and Lamin A/C with previously published data which led us to investigate further and evaluate whether there could be other possible players at the promoter level in this A‐T cellular model. Recently, in the same cell types, we focused on the dex‐mediated modulation of the pRB, Lamin A/C, E2F1, and Lap2α interactions [19], and therefore, we decided to re‐probe these targets together with FoxO3a on the p21 promoter (Fig. 6). The transcription factor E2F1 was tested because the binding site of the *CDKN1A* promoter is adjacent to the FoxO3a one, and it may regulate p21 expression. The factors FoxO3a and Lap2α were specifically increased by dex, in the *CDKN1A* promoter, only in AT 648 hT cells, while pRB protein was incremented in both cell lines after dex. The transcription factor E2F1 was decreased only in AT 648 hT cells after dex stimulation, thus supporting the suggestion that it was not responsible for p21 expression. From these results, we can conclude that the transcription of p21 is regulated in some way solely by FoxO3a, but this result is inconsistent with previously published data which states that not only HDAC2 should inhibit the expression of the target gene p21, but also the presence of pRB should have an inhibitory action on *CDKN1A*.

## Discussion

Ataxia elangiectasia is a multisystem disorder where patients are affected by neurodegenerative disorders, ataxia, oculocutaneous telangiectasias, immunodeficiency, and radio sensitivity and are at a higher risk of cancer. No cure is available at present, but glucocorticoid administration in A‐T patients has led to beneficial effects and an enhancement in quality of life [11, 13]. Dexamethasone administration in A‐T patients by autologous RBCs has reached phase 3 of a worldwide clinical trial (NCT02770807) with promising outcomes. Further investigations into the exact molecular mechanism of action of dex in A‐T are necessary so that targeted therapies can be developed. We previously observed that dex is capable of selectively modulating A type lamin dynamics in A‐T [19]. We now report an unusual behavior that somehow regulates p21 gene expression in a different manner to that which is reported in the literature [21, 27].

Firstly, we discovered that dex can modulate the Lamin A/C‐HDAC2 interaction and reduce A‐T amounts to those found in WT by PLA assay. This pattern was also confirmed by Co‐IP assay, the conventional method used to verify protein and protein interactions. However, the latter technique could lead to the presence of false interactions [28], and it is not capable of detecting labile or transient interactions [29]. Total amount of HDAC2 and Lamin A/C was also evaluated. A‐T cells revealed more HDAC2 than WT cells, whereas the same quantity of Lamin A/C was found in both cells, while dex did not seem to significantly modulate HDAC2 quantity.

Secondly, due to the involvement of the above‐mentioned interaction in the A‐T dysregulated p21 expression, HDAC2, and Lamin A/C levels on the FoxO3a binding site of *CDKN1A* promoter were also evaluated. Surprisingly in A‐T cells, HDAC2 does not have an inhibitory effect as previously reported in other cell types. In fact, only in A‐T cells after dex addition does the accumulation of HDAC2 in the p21 promoter region correlate with its incremented expression. Also, Lamin A does not seem to be necessary to recruit HDAC2, as demonstrated by Mattioli *et al*. [21]. These data suggested that at least in A‐T, HDAC2 does not have a role in its deacetylase activity and histone regulation [21], thus avoiding the transcription of *CDKN1A* gene. Some other molecular pathways must be responsible for promoting p21 overexpression after dex treatment. In this circumstance, E2F1 seemed to be uninfluential as its binding to *CDKN1A* promoter was inhibited by dex in A‐T. The only evidence is the elevated amount of FoxO3a bound to the promoter, which is capable of transcribing p21 in dex‐stimulated A‐T cells.

pRB levels increased in all dex‐treated samples; thus, its inhibitory action is probably unrelated to FoxO3a activity, and pRB may be associated with some other proteins present in the recorded complex. Another difference related to increased p21 expression, apart from FoxO3a, is the large amount of bound LAP2α index‐treated A‐T cells. LAP2α has an important role in Lamin A/C solubilization and chromatin regulation [30, 31, 32, 33], and it is possible to assume that somehow LAP2α could evade the HDAC2 inhibitory action or promote FoxO3a activity. The role that we suggest for LAP2α has been previously noted by Ricci *et al*. [19], where it positively regulated the THBS1 expression after dex treatment in A‐T cells, even if in a slightly different factor composition. Also, the role of Lamin A type should be considered, as it decreased after dex action in A‐T, and therefore, its inhibitory function disappears. Additionally, a double situation was found in the *CDKN1A* promoter neighborhood with the inhibition of E2F1 complex by HDAC2/pRB [34] and the simultaneous FoxO3a stimulation by LAP2α and consequent p21 expression.

The data here reported demonstrated that dex can induce a differential biological behavior between WT and A‐T cells, in particular concerning the control of the Lamin A/C‐HDAC2 interaction and the emerging role of Lamin A/C as a regulator of chromatin dynamics and as a local controller factor of genes involved in cell fate. In A‐T, p21 expression was dysregulated, and consequently, its cell cycle surveillance was impaired.

This investigation concerning p21 promoter effectors, clarifies how dex can indirectly modulate p21 to restore it to WT levels. The results of this study help to explain the mechanism of action of this unique drug currently undergoing phase 3 clinical trials and provide support for its future use in patients.

Furthermore, since p21 is dysregulated in some tumors [35], it could be interesting to further investigate the action of dex on the multicomplex protein assembly Lamin A/C, HDAC2, LAP2α, pRb, E2F1, and FoxO3a on the CDKN1A promoter that might regulate p21 expression, in some cancer cell types.

## Conflict of interest

Mauro Magnani holds stock ownership in EryDel S.p.A. None of the other authors have competing interests.

## Author contributions

MMe initiated the project. AR, FB, and GM contributed to the material preparation, data collection, and analysis. AR and MMe wrote the first draft of the manuscript. MMa contributed to the revision of the paper and financial support. All authors contributed to the study conception and design, read and approved the final manuscript, and commented on subsequent versions of the manuscript.

## Data Availability

All data generated or analyzed during this study are included in this published article and available from the corresponding author on reasonable request.
